# American Academy of Dental Science

**Published:** 1895-11

**Authors:** 

**Affiliations:** American Academy of Dental Science


### AMERICAN ACADEMY OF DENTAL SCIENCE.

THE regular meeting of the American Academy of Dental Science was held at Young's Hotel, Boston, February 6, 1895, at six o'clock, President Smith in the chair.

The paper for the evening was by Dr. R. R. Andrews, of Cambridge; subject, "A Contribution to the Study of the Structure of Dentine."

(For Dr. Andrews's paper, see p. 655.)

### DISCUSSION.

*President Smith.*—We have listened to a very interesting paper, and it has certainly been a great pleasure for us all to witness the beautiful slides which have been shown here to-night. The essayist of the evening referred particularly to Professor Bates. Perhaps Professor Bates will open the discussion for us.

*Professor Bates.*—Dr. Andrews referred to Dr. Rose's work in Germany, speaking of him as one who has done more, perhaps, than anybody else of late years in this histological work. He did not mention the fact that Dr. Rose is a practising dentist, and I might say that the particular line of work in which he has made his investigations is in regard to the evolution of the tooth, and I suppose the work he has done in that line is, perhaps, the best that has been presented to us. He has a theory of his own concerning the evolution of the tooth which, perhaps, contradicts the theories of our American workers in that line. If you have followed the subject at all, you know what our theories of tooth evolution are. Of course the air is full of evolution nowadays. There is scarcely a magazine we pick up but contains something on the subject, and everything, particularly in the field of biology, seems to be based upon evolution, and we hear it everywhere, so that the tooth comes in for its share. Professors Cope, of Philadelphia, and Osborne, of New York, are the men who have done the most in this country bearing on this question of evolution. These gentlemen are both palæontologists and have approached the subject from the stand-point of the palæontologist.

Dr. Rose has taken the matter up in a different way; he has approached it from the stand-point of the biologist, and in his histological work he has tried to show the different stages through which the original reptilian tooth, which was the progenitor of all teeth, has changed as it has been called upon to do more severe and varied work, so that the six-cuspid mammalian tooth represents six primary cones which have merged together. Now, that theory has been treated by Professors Cope and Osborne in this country, and while they claim that this mammalian has descended from the reptilian tooth, as Dr. Rose does, at the same time they claim that this evolution has come about by the production of additional cusps on the original central reptilian cone. So, then, these are the two theories of tooth evolution which are presented to us to-day, and the great battle is being fought along these lines. The subject which Dr. Andrews has paid particular attention to, the sheath of Neumann, is, like the membrane of Nasmyth, something we really know very little about. I think Dr. Andrews has shown us to-night very clearly the existence of such a tissue, and that we cannot but feel that statements to the contrary are simply what we might expect from those men who have not had advantages equal to those of Dr. Andrews and the workers he has quoted.

*President Smith.*—Gentlemen, the society always listens with interest to whatever our honorary member, Professor F. W. Putnam, has to say when we have the pleasure of his company at our meetings.

*Professor Putnam.*—This subject is so entirely out of my line that I do not know just how I can add to the discussion. I wish to say, however, that I have been instructed every moment while listening to Dr. Andrews, and his illustrations have exemplified the truth of a remark I made when I was last with you. I said that I thought it was necessary for a dentist to have a very delicate touch in order to amount to anything in his profession. Perhaps not all of you are familiar with the working of the microscope and the making of such sections as we have seen this evening. I have seen a good deal of that work carried on, and I know that the dissecting out of one of those little cells is an exemplification of my ideas in regard to delicacy of touch. Those of you who have done such work know how extremely careful one must be, how the least unsteadiness will destroy all your work of hours in cutting your section and then picking with a fine needle to clear the little cell which you wish to show. The sections which have been presented to us to-night must represent hours and hours of work. It is certainly very beautiful work, the making of those sections; and the sections themselves have given to us important information in regard to the formation and structure of the tooth. I have never seen anything surpassing these histological specimens. As to the subject-matter itself, that is entirely out of my line of research.

*Dr. Clapp.*—I would like to ask Dr. Andrews if he has discovered anything in the structure of dentine, after all the time and work he has spent to get this subject in shape, that would lead to any theory which would be of practical value to us in the treatment of sensitive dentine? Now, in decalcifying these teeth and preparing them for his slides he finds that certain things perform certain functions, and some portion of the tooth conveys sensation, and I would like to know whether, from the data he has collected and from his experience in the treatment of dead teeth, or the teeth that he works on, he has formed any theory which will be of practical benefit to us in our operations or teach us what to use in the cavity of a live tooth to reach the sensitive portions?

*Dr. Andrews.*—I wish I could answer that question in the affirmative. It opens up a line of thought that is likely to involve a great deal of research before anything like a satisfactory answer can be given. There is no question but that the pulp is affected by the action of acids on the dentine and by other causes, but we do not as yet know in what way. Tomes speaks of a specimen where the nerve-fibre terminations are shown over the large ends of the odontoblasts. It has never been proved that the nerve-fibres enter and go along the fibrils. It would seem as though the sensation is conveyed by the fibril to the pulp, but in what way is still a mystery. There is something singular to me in this fact; that in opening up a cavity in the crown of bicuspids and molars we proceed with the drilling without giving much pain, until we come to the cavity proper—a most intensely sensitive spot—somewhere between the enamel and the pulp. Well, we cut through this and we can then go deeper if need be without much sensation. Now, the question is, Why should the fibrils be so sensitive at that particular point? I have reasoned that it is the action, probably, of the acid on that softened mass, all the fibrils within it are disturbed, but when we cut through that mass we come again to the single fibrils in the normal basis-substance, and the sensation is very much less. That is the only way in which I can get any intelligent explanation of the intense sensitiveness we sometimes meet with in preparing cavities. Just how sensation is carried to the pulp I do not know, nor do I know just how to certainly obtund it from any observations that I have made.

*Dr. Clapp.*—In regard to sensitive dentine, I had yesterday an experience that was both interesting and provoking. The patient has complained of pain in the two right superior bicuspids on mastication. I examined the teeth, and with an exceedingly small, sharp-pointed exploring instrument I could just get it into a depression in the second bicuspid. It would not go in more than the one-hundredth part of an inch and it was as fine as a needle, but the sensation was excruciating and caused the tooth to ache for a long time. After putting on the rubber and drying out the tooth, I was enabled to get in just a little way and make a very small cavity, enough to hold a speck of gold. Now, I didn't learn anything from that operation excepting that a very small thing can be exceedingly painful. I think it was the smallest crown cavity I ever saw that was sensitive.

*Dr. Andrews.*—In that beautiful section of Dr. Gysi's showing the cross section of the sheaths and the same sheath in longitudinal section, all through the structure of the matrix are seen faint processes, which he calls "gelatin-yielding fibres." You remember that I spoke of a fibrous substructure on which the gelatin has been deposited before the calcoglobulin is given off from the odontoblasts. This fibrous substructure was demonstrated three or four years ago by Mummery, of London, and described and illustrated, and these appearances that are seen all through the tissue I have shown are spoken of as gelatin-yielding fibres of the dentine; not the fibrils or their ramifications. And it is a question in my mind if, in what we call the Mummery fibres, upon which this tissue is built, there may not be a trace of gelatin left in their structure. I do not believe their is a living reticulum through the basis-substance of the dentine other than the ramifications and anastomoses of the fibril within the canaliculi.

*Dr. Clapp.*—Just one word more in regard to sensitive dentine. Of course, if we knew entirely the structure of dentine and all about it, we could perhaps do more than we can now to overcome its sensitiveness, but it seems to me that this sensitiveness is caused by pressure,—not by the act of cutting. I now make it a point in cutting sensitive dentine to have very sharp instruments, so that the pressure in the act of cutting may be as slight as possible.

*Dr. Andrews.*—This may be a little off the subject, but I would like to ask if any of the members have used glycerin and cocaine, mixed together, to allay the sensitiveness of teeth. The glycerin has the power of absorbing moisture and the cocaine would act on the fibres. I have tried it and in some cases it works very nicely.

WILLIAM H. POTTER, D.M.D.,
*Editor American Academy Dental Science.*

---

THE regular meeting of the American Academy of Dental Science was held at Young's Hotel, Boston, March 6, 1895, at six o'clock, President Smith in the chair.

The paper for the evening was by Dr. Frederick Bradley, of Newport, Rhode Island; subject, "Judgment an Important Factor in the Preservation of Teeth."

(For Dr. Bradley's paper, see page 664.)

DISCUSSION.

*Dr. Cooke.*—I congratulate the essayist in showing good judgment in writing his paper. If a man has judgment he has something which education will not give him, and which training cannot take away from him. Information and manipulation he may acquire by patient study, but if he has not the attribute which is sometimes called "horse sense," no amount of education will put it into him. Judgment is required in other persons as well as dentists. It seems to me that patients should exercise good judgment when they select their dentist. A certain man who lives out of town once told me that he always had his teeth cared for in the city, as he didn't have much faith in the dentist in his town. But as the local dentist was convenient he could send his children there very easily. I could not help thinking that possibly this convenience might be a cause for regret when the children grew up. Much more depends upon the care of young teeth than upon after-treatment. Parents, when they come with their children, should exercise proper judgment, and bring them at a reasonable time, and also insist on proper treatment when they do come. In some cases you cannot do much for the children, especially if they have not been trained to behave when they are at home. Some of them have been petted and allowed to have their own way, and they won't mind you any better than they do those at home, and when you get one of those hard cases the best that can be done is to carry out Dr. Stebbins's idea of taking off the rough edges of their cavities and wiping them out with nitrate of silver.

In regard to adult patients, it seems to me they should have good judgment in yielding certain points, and in their appreciation of what has been done. They come to us and there are certain things to be done; their mouths are in a very bad condition from neglect, and it takes considerable time and skill to get them into shape again, and if your charges are high very likely they go to somebody else, who reaps the benefit of the good work you have been doing for them. There is neither judgment nor justice in such cases.

In the selection of our methods, materials, and instruments, we should be very careful to get the combined judgment of the profession on any one thing before we adopt it. When amalgam first came into use it was supposed to serve the purpose just as well as gold, and was much easier to put in. Then came the reaction, and for a while it was not used at all. We had the same experience with crown-work and in the use of gold; because one piece of gold would stick to another, many tried to build up monuments on any part of a tooth. But they soon found that a structure was no stronger than its foundation, and the result was a reaction in the use of cohesive gold. Plastic fillings and bridge-work have been through the same routine, and so has copper amalgam. Our experience in all these things proves that it is best to go slow in accepting a thing merely because somebody claims to have had good success with it; the favorable experience may have been a matter of chance. When you get the combined judgment of all, then it is safe to use a preparation or method.

One of the things which greatly tries us is the number of failures that are made in orthodontia. I know of two cases where the patients have been induced to allow the extraction of two teeth,—in one case they were the bicuspids, and in the other case the bicuspid and the first molar,—and in both cases a result was produced by which the patient was handicapped for life. It seems to me in a position of this kind a man shows very poor judgment in trying to settle everything for himself. There is no need of it where we have such friendly associations that a man can very easily obtain *sub rosa* information,—if you wish to call it that,—and the patient need not know that you have consulted anybody. We have to be very careful about consultation, for if we call another dentist in consultation the consulting dentist may get the patient, and that ends the case so far as we are concerned.

I think we see about as bad failures and evidences of misplaced confidence in the matter of bridge-work as in any line of dentistry.

I remember seeing an extensive bridge which was put in by some advertisers in New York or New Haven, and it failed. Of course the patient felt very badly about it, after spending money and time on it and supposing that it was going to last a lifetime. When putting in any extensive bridge-work I have been in the habit of telling the patient that the appliance will last a certain length of time, but that eventually it will be necessary to have a plate. The bridge is simply a convenience which will put off the necessity of wearing a plate as long as possible. I saw a case where a man extracted two laterals and two centrals, more or less decayed, so that he could put in a nice bridge, running from cuspid to cuspid. You would expect that a man who had the courage to attempt such a thing as that would have the manipulative ability to do the work decently and to make an appliance that could be worn with a minimum amount of discomfort by the patient. This was a miserable failure.

There is one point which the essayist touched on with regard to the education of the dentist which I think is very important. I refer to his remarks on the necessity of trying to bring the student to think for himself. It seems to me that is one thing we have left out in our school instruction. The student may be able to answer the questions in his text-books, he may be able to manipulate well, but if he does not know just why he does a thing, the instruction he gets is not of much practical benefit when a complicated case is met.

Another place for the exercise of judgment is in the length of operations. Where patients have been subjected, as they have been in the past, to operations extending over three, four, or five hours, it is a hard strain on the mind, and brings the patient into an attitude towards the dentist that is anything but pleasant, and they remain away so long that the good result which you may have accomplished is destroyed.

It seems to me that long operations have done a great deal of harm to us as a profession, and I think that the day has gone by when a dentist should attempt a large amount of work at one sitting.

*Dr. Potter.*—In the use of good judgment in dental operations much depends on a thorough knowledge of the conditions under which we operate and the purposes for which we operate. We must understand the medical side of our case first of all, and then the mechanical side.

If we have this perfect knowledge we are not likely to go off in an erratic course. If we see a demonstration of a certain method, like that, for instance, which was given by Dr. Libby a short time ago with regard to burnishing gold, we do not necessarily either adopt it completely or reject it, but we are more likely to find it useful in certain parts of our work and yet not serviceable in others. I can imagine that if that demonstration was given to students, many of them would at once decide that it was either good for nothing or that it was the only way out of a difficult piece of work. The proper effect which any unusual method of practice is likely to have upon a well-educated mind is to afford certain suggestions; it will not revolutionize our work, but will help us to do it better.

*Dr. C. H. Taft.*—I do not understand the purport of Dr. Bradley's paper to be either that of commending or of condemning any special way of practising dentistry. A very great deal has been said in the past against copper amalgam, and in so far as it has a bearing upon the subject of the saving of teeth, I would like to take exceptions to some statements that have been made. You all know what my views are in regard to the use of amalgams. It is rarely ever that I use amalgam when I can substitute anything else as well. It seems to me that our profession shows a lack of good judgment, if we are going to use amalgam at all, in the wholesale manner in which silver alloys are used to-day and have been in the past.

I am very sure that where I have had occasion to take out one copper amalgam filling I have had to take out or patch up twenty-five of so-called silver fillings. Since I have been away for the last two years I have not seen many of my copper amalgam fillings, but in the previous years of my practice I had the best of results with copper amalgam as a preserver of tooth-substance. I have, however, quite recently seen some of my copper amalgam fillings in the mouths of three of my patients that were inserted four or five years ago. There is absolutely no shrinkage whatever, and the teeth are in a perfect state of preservation. If we are to show good judgment in any particular way in our efforts to save teeth, and if we must save them with amalgam, I cannot for the life of me see what the objection is to copper amalgam, and I wish the gentlemen who do oppose it so strenuously would tell me wherein I fail to get satisfactory results. I certainly have had the very best results with it, and I should not be ashamed to have any of the gentlemen of this society, or of any other, see the fillings that I have inserted, and let them judge for themselves whether or no these fillings are preserving the teeth, whether there is any shrinkage or leakage, and whether they have not just as smooth a finish as any of the gold fillings that I have ever inserted. With my own experience in mind, I have always endeavored to find out wherein copper amalgam fails in the hands of so many men, and why it is that they are so constantly casting slurs upon it. I am not speaking now of what may be its injurious effects upon the general system, but rather of its utility as a filling-material.

*Dr. Williams.*—There is a point in regard to the fact last alluded to by Dr. Taft which would seem to indicate that proper judgment in operating would contraindicate the use of copper amalgam in the interior of a cavity in a tooth. The greatest density of a tooth, of course, is the enamel, and it would be perfectly proper to make a hard, flinty surface for grinding purposes; but to make a hard filling for the interior of the tooth, in my observation, has often proved to be destructive to the vitality of the tooth. My theory is that we should have something for the interior of the cavity that corresponds somewhat,—certainly not harder than the dentine at that part of the cavity. There is also a mistake which is very commonly made,—that is, in filling up those cavities with phosphate cements, which in a short time become very hard and rocky, no elasticity, no porosity, no free play for the natural pulsations in the circulation of the pulps, and they rebel after a while; and frequently I have found pulps destroyed from that very cause, from having too hard material in their vicinity.

*Dr. Cooke.*—I will try to answer Dr. Taft's question in regard to copper amalgam. I used quite a good deal of it at one time, and for a while was strongly in favor of it, but I soon began to notice that people who had copper amalgam fillings had very sensitive teeth, and, later, I found cases where decay occurred around the copper amalgam at the cervical margin. The fillings were dished out so that you could put your instrument in between the wall of the cavity and the filling. This seems to be the testimony of nearly every one who has used copper amalgam, and Dr. Taft must have had a very peculiar experience to be so in favor of it. I have inquired at the dental depots regarding the sale of copper amalgam, and they say that where they used to sell a great deal of it the sale now is very small. I admit that I have had some cases in which the conditions seemed favorable to its use, and it appeared to preserve the tooth in a remarkable degree. I should think if Dr. Taft, holding the views that he does, desired to use any amalgam, he would prefer one of the old kinds that does not wear. How he can use copper amalgam, when there is such an evaporation of mercury from the surface all the time, I cannot understand. I should think he would be afraid that it would poison his patient.

*Dr. Taft.*—My experience has been just the opposite of Dr. Cooke's. I have used copper amalgam in places where the conditions seemed most trying, perhaps on the buccal surfaces of molars and bicuspids, and in cases where the teeth were affected with white decay, and in no case that I can remember has it been necessary to do any subsequent patching about the margins of the fillings. I have been surprised in taking out copper amalgam fillings where two-thirds of the tooth, perhaps, consisted of amalgam, and where, in preparing the cavities, there had been absolutely no undercuts or retaining-points made, to see how those fillings have remained in place where no amount of force employed in the process of mastication had caused them to break away or to come out. I wish those gentlemen of the profession who are continually objecting to copper amalgam could look into the mouths of some of my patients and see the fillings that I put in several years ago. It would be the best answer in the world to these arguments that are being urged against this agent, and is, to me, good evidence that I preserved the teeth of my patients successfully with copper amalgam before I had practically discarded the employment of any sort of an amalgam as a filling-material.

*Dr. Werner.*—You don't use it now?

*Dr. Taft.*—Not when I can serve my purpose with something else.

*Dr. Andrews.*—How about its washing out?

*Dr. Taft.*—That is a thing I wanted to speak of. I have seen copper amalgam fillings that have dissolved possibly just as much as a gutta-percha or a cement filling would; or, to be more accurate, not exactly dissolve, but, rather, in course of a few months, become cup-shaped; and I believe that the cause of that is the leaving in of too much mercury. You will find that when you first mix up the amalgam it makes a glistening mass that is exceedingly moist. It is my habit to place it in a piece of chamois-skin or a napkin and squeeze out all the mercury possible. In reheating, you will express still more mercury, and after further continued heatings you will get very little out of it. When that amalgam is placed in a tooth you will have the densest kind of a filling possible, and one which will become as hard as steel. I believe if you do not squeeze out all of the mercury, then you are likely to get fillings which will possibly become cup-shaped in the course of a few months, though they do not draw away from the walls of the cavity as Dr. Cooke describes.

I have also noticed that if the filling is inserted immediately after the first heating it will set very quickly, but after two or three heatings it will take half or three-quarters of a day before it sets. By taking your copper amalgam, heating and grinding it up thoroughly in a Wedgewood mortar, and manipulating it in the way I have described, you will have obtained a filling that you need not be afraid is either likely to disintegrate or fail under any condition whatever to stand the test of time.

*Dr. Andrews.*—There is one authority on copper amalgam fillings who says that he uses the amalgam just as it is. If he uses it just as it mixes, without squeezing out any mercury, he always has success, and if he presses out the mercury he always has failure. It merely shows the difference of opinion among different operators.

*Dr. Daly.*—There is one thing I have noticed about copper amalgam fillings,—those that stay entirely black are permanent ones, and those which have a polished, glistening surface are the ones that dish out. I don't know whether the black ones contain the least mercury or not.

I would like a dental definition of the word "permanent" from some of the members present. There seems to be a difference in the meaning of this word as used by different people. Some seem to imply that a gold filling is the only permanent filling, while it is claimed by others that cement is permanent because it lasts up to a certain period, and then must be renewed with another cement filling, which is permanent for another period. The term is also used in speaking of bridge-work. The essayist spoke of putting a full denture on four teeth. I know of a case which was placed on in that way that lasted for fifteen years, and in the judgment of the operator putting them on in that manner they were "permanent." The teeth supporting the bridge were destined to have been lost at the end of fifteen years with or without a bridge (for if not by a bridge they would have been destroyed by a plate), and if the bridge was made to do service during the life of the supporting teeth, then it was a permanent denture. It was all that lay in the power of the dentist to do, and its chief value was in the fact that the patient was saved from the disagreeable features of a plate for a considerable length of time.

*Dr. Werner.*—Another objection to the copper amalgam is the discoloration. I am one of the fortunate ones that never used it. I have found no need of it in my practice, and a decided disinclination on the part of my patients to have copper amalgam or any amalgam put into their teeth.

If it were not for this constant lack of judgment medicine would be nearer an exact science, and dentistry would be still nearer an exact surgical and mechanical science. Is the man living that has the exact judgment and foresight to tell what is going to be the very best thing to be done in every instance? It is not possible; it is simply possible to come near it in the majority of cases. The one who puts in temporary fillings in places where he knows they will have to be soon replaced, does he use good judgment from the financial stand-point of his patients and from a scientific stand-point of his professional calling? I think he lacks good judgment, lacks skill, perhaps, and to a degree abuses the confidence the patient places in him. Many bicuspids are sacrificed every year, and in the course of three or four years the operator can see that judgment was lacking when he cut those bicuspids off. And what was the factor that induced him to do this? Perhaps lack of skill to put in a contour gold filling, or perhaps he allowed his patients to grow up with an absolute horror of an hour or an hour and a half's sitting. Too many patients have decided ideas that a dental operation must be exceedingly short, gentle, and easy, and many operators try to please them too much in that respect. That is not only bad judgment, but dishonesty to patients and a lowering of professional morals. As Dr. Cooke said, I do not believe in requiring your patient to stay in the chair for five hours,—that is much too long.

I remember a distinct case of a boy, whose deciduous molars were decayed, where I was obliged to fill cavities with cement, because his step-mother decidedly instructed the nurse who brought him that no gold should be put in his teeth. Now, those simple cavities could have been filled with gold, without the use of the rubber dam, in the same length of time that it required to put in the cement, and the fillings would have lasted until he was nine or ten years old, but the cement fillings will all need replacing before he loses the deciduous teeth. The essayist has covered almost the whole field of dentistry in the word "judgment." I am glad that our calling has enough of the surgical and the mechanical character to make nearly all our operations a success. Happily, we do not have to rely upon the uncertain action of any drug, either in large doses or in the one-hundredth or one-thousandth part of a drop. We apply surgery and mechanical art, which comes nearer to being exact than any other treatment in the medical profession.

I remember one evening I went home from a meeting of dentists with a brother member. On the way he was speaking of the virtues of copper amalgam. When we reached the corner of the street where we parted I informed him that I never used copper amalgam. He was perfectly surprised; he did not see how I could get along without it; he had saved more teeth with it than with anything he ever used. Now, his love for copper amalgam was brought about from his lack of skill in using gold, for little or no skill is required in using amalgam or the plastics. You must have a good degree of skill, a certain amount of common sense, good judgment, and a standard for honesty to do the best for our patients. You must be on the conservative side, yet at the same time listen to the enthusiast. It is the enthusiast who has brought our calling to what it is: in the skill of operating, the inventions of instruments, the ingenious devices such as bridge-work,—and who doubts the value of bridge-work? I have a distinct case in my practice where on four teeth, two upper molars and two cuspids, an entire upper denture is supported with the utmost comfort. Nothing could be more beautiful, more cleanly, more satisfactory, than this device to assist in mastication of food and in the restoring of personal comfort and appearance.

The one who simply puts in fillings, and never thinks of talking to his patients about cleanliness and the use of the brush, does not use good judgment. In addition to the cleanliness required to prevent the decay of the teeth, the gum needs the massage treatment of the tooth-brush to promote its health.

There are many other places where the dentist shows a lack of judgment,—for instance, in not restoring to full contour approximal surfaces. We see many cases of separation,—spaces left between the teeth or flat fillings that allow food to crowd in, producing uncleanliness, inflammation, and discomfort, and, in three or four years, redecay of the teeth. Such want of skill and judgment is what has put gold operations in disrepute. A properly shaped and contoured gold filling will secure exosmosis, suction, the cleansing produced by deglutition, talking, etc., and will be of immense value in keeping the teeth clean and free from further decay. Many operators have failed because they did not know the value of full contour-work, and it is from these failures that we should learn.

*Dr. Daly.*—And then the question arises, Is your second judgment, after the first failure, any more successful than your first judgment, before failure? That, of course, remains to be proved.

I did not quite see the consistency in Dr. Werner's saying, in the first part of his remarks, that he did not believe in allowing patients to grow up with the idea that dental operations should be easy, gentle, and short, and then stating that he did allow a patient to influence him to make the insertion of a filling easy and short. The most influential suggestions that we receive are from our patients themselves.

What gentleman present would not rather put a gold filling in a first bicuspid, a large part of which is decayed, than an amalgam filling which the patient demands? Such fillings have not always been successful, but the patient does not usually tell the history of the filling when she goes to another dentist and presents some work that has failed. Does she say that she was the biassing element that influenced the dentist to do the work the way he did? Oh, no; she never says anything about that part of it, and so the dentist gets the blame for the failure, while the patient was at fault. Many people jump at the conclusion that Jones, Smith, or Brown has poor judgment, because they have seen a piece of work that has failed, when really the patient has been the one at fault. The mass of dentists suffer not a little from this misunderstanding of the facts in any given case. The dentist who has a particularly intelligent clientele, of course, does not suffer so much.

*Dr. Werner.*—It was not a lack of judgment on my part that made the case I mentioned a source of regret to me, but a positive lack of judgment on the part of the boy's mother. It would not have hurt the boy more to put in the gold fillings; it would not have taken longer, as they were the simplest crown cavities, and had they been filled with gold there would have been no necessity for further work until the teeth were lost.

I have an admirable case that will cover the matter that Dr. Cooke spoke of,—two daughters in a family that I treated. They came to me with the shabbiest kind of dentistry. Their teeth were extremely sensitive, and they were on the brink of artificial crowns and dentures. I treated them extensively, and did the best I could for them. I wish now that I had only done one-quarter as much, for when I came to render my bill, amounting to several hundred dollars, the father was astonished and indignant, and said they had been with the previous dentist for years, and he had never charged more than fifteen dollars for one series of sittings. I had done the difficult work necessary to put their teeth in order, to save them for ten or fifteen years. The next operator will get the benefit and credit of my work, in a measure at least, and I get the blame for being unreasonably high in my charges. Now, there is a case where you might charge lack of judgment. Perhaps I was a little too enthusiastic, too conscientious in my work, yet my very best judgment told me to do all that was necessary for the preservation of the teeth; but, in the light of after-events, I would probably not have lost the patients had I done but one-third as much. Use your best judgment in each individual case, study it from all its standpoints, and do the best you can under the circumstances.

*President Smith.*—In the last case that Dr. Werner spoke of I think he showed good professional judgment, but a lack of business judgment. What he ought to have done in that case was to have called the father of the family to his office, told him what ought to be done, and about what it would cost; but in going ahead and doing a great deal of work to put those mouths in order, without knowing whether the fee charged would be satisfactory, he showed a lack of business judgment.

I think it is the experience of many of us that, if a patient comes from a brother dentist presenting some case which gives evidence of a lack of judgment in its treatment, and the patient makes no explanation whatever, the dentist who receives that patient feels astonished that Dr. —— should do so and so. As Dr. Daly has put it, we do not know the circumstances, and should be very careful about offering any criticism. Many of us have patients in society who are up all night and sleep most of the day. They are nervous, irritable, under the care of the doctor, and constantly taking drugs. Yet their teeth must be saved. They are sensitive all over; their nervous system is entirely demoralized, and they are totally unfit to withstand any dental operation. I had a patient in my chair today who is a typical example of this class of persons, and if I could charge enough to drive her out of my office I would do so; but she has never found fault with my bills, so I have to do the work, though it is exceedingly trying on my own nerves to do it. If the teeth were of good texture I could separate the molars and bicuspids and put in contour fillings, and they would last during her lifetime. But all I can do with this patient is simply to excavate decay, and, on account of the sensitiveness of the dentine and the nervousness of the patient, it is a most difficult task with the sharpest burs, with all the obtundents and greatest care, and I am obliged to spend two hours where I should spend one. Now, in that case I have put in gutta-percha fillings, and in some cases built those teeth right together solid, and they have been kept along for years in that way. Who can do better under such circumstances? Could better judgment be shown in the treatment of those teeth? I have had patients who, after a long struggle, have finally made up their minds to submit to an operation of two and one-half hours and have a contour gold filling put in; then, after that, there would be a collapse, a reaction, and the question confronts us, Is it good judgment to put those patients through such a course as that? Have we any right to ask our patients to undergo treatment that disturbs their vitality for the sake of the preservation of their teeth with gold? We must govern our judgment by the patients and the conditions which we meet.

I was amused as well as instructed in reading an article by Dr. Perry in the last INTERNATIONAL DENTAL JOURNAL. He spoke of seeing a large gold filling in a bicuspid that was somewhat top-heavy with its gold. He said that filling, put in in such a way on such a tooth, could not last more than two years, or three at the most, and then he says, "How much better judgment it would have been to put in a cement filling;" and this is the part that amused me, because the title of his paper was "Moderation in Practice and in Statement." How much better judgment it would have been to have put in a cement filling, which would only have taken ten minutes, and could be replaced whenever necessary at an expenditure of five minutes more. If Dr. Perry can properly excavate, dry, and put in a cement filling in ten minutes, he can do much better than I can.

Regarding the matter which the essayist referred to, and which has been touched upon by Dr. Daly, about the suggestions of patients, I think we *are* governed somewhat by the suggestions of patients, but at the same time I think there should be a limit to it. Now, I have heard men make the statement that they would not listen to any dictation from their patients,—if the patient did not think that what they proposed to do for them was just what they wanted done, then they could go somewhere else. I think that is a radical statement, because if you have patients who are treated by homœopathic physicians, and from those physicians receive the idea and thoroughly believe that an amalgam filling is injurious, it is absurd for a dentist to insist on either putting amalgam into their teeth or nothing at all. He can preserve teeth temporarily with gutta-percha or cement, and if they have to be repaired every three or four months, that is their lookout. You can tell them that amalgam is the very best thing you can use under the circumstances, and if they say, "I object to amalgam on account of its being injurious," you have no need to endorse that judgment, but go ahead and use the material which will best serve the purpose, amalgam excluded. I think it is perfectly proper to waive your point under such circumstances as those, but when it comes to a case of a woman demanding that an entire gold crown be put on a first bicuspid, with a diamond set in, as I have seen done, then I say it is time for the dentist to stand on his dignity and say, "I will not do it," and let that patient go if she chooses. There is a case in my practice that is a good illustration of what may result from accepting of suggestions from patients. A lady went to a certain dentist in this city, and in the course of his work he found that the pulp in one of her teeth was dead, and he decided to remove it. As he was drilling into the pulp-chamber the patient stopped him and said, "Here, I know a friend who had a tooth ruined by having it drilled into." The patient was of a good family and very positive in her opinions, and so the dentist listened to her arguments and temporized with the tooth. He knew better, because he is a good dentist, but still he did not want to offend the patient by insisting on doing what he knew to be right. He had not removed the pulp from the pulp-chamber when he was told to stop, and he gave the tooth the best treatment he could under the circumstances and closed the cavity. As might be expected, an abscess formed, and this he attempted to treat by the use of antiseptic washes, but of course as long as the foul pulp remained he could not obtain a cure, and the matter was carried along this way for a year, and she never would permit him to go into the roots, because she knew it was wrong. Finally the patient came to me with this tooth. As I took out the dressing she asked me if there was any reason why a cure could not be effected, and I replied that I could see no reason, and went to work to do just what he had tried to do. She put a stop to my doing it, saying she didn't propose to have anything of that kind done to her tooth. I immediately arose in all the splendor of my dignity, and said that I should either treat that tooth as I wanted to do or she could go somewhere else. So shocked was she at being spoken to in such a manner that she absolutely cried like a baby in my chair. The sight of the tears made me feel repentant, and I began to explain the case to her, and drew a diagram to illustrate. She allowed me to treat that tooth, and it was cured. She told me when she first came who had treated her, and when I looked at the tooth I was thunderstruck to find that man's name coupled with the treatment of this tooth. I said nothing, however, and when we came to the discussion as to whether I should drill into the tooth or not, the lady said, "That's just what Dr. —— wanted to do," and I then saw through the whole matter at once. He knew what ought to have been done, and he did as well as he could under the limitations placed upon him, and the only mistake he made was in not insisting on doing what he knew to be right or nothing at all. The lady and some others of her family have become regular patients of mine, and he has lost them simply because he did not stand up and resolutely say, "I must treat the tooth in the proper way or not at all." If no one else has any remarks to make I will ask Dr. Bradley if he has anything to say before closing the discussion.

*Dr. Bradley.*—I think the remarks generally agree with the opinions expressed in the paper. The point brought out by our President and Dr. Daly I think I emphasized quite markedly, and it may do no harm to repeat what I said in my paper, that there are cases where it seems to me it is proper to accept suggestions from the patients, and in other cases it is perfectly legitimate to use your own judgment, and do what in your opinion the case demands, even though it is not just as the patient suggests. In regard to copper amalgam, for those who are satisfied with the results obtained in their practice, I should say it was good judgment to use it; I have used it a very little myself. I will say that I do not use it now, as I like the appearance of other amalgams better, and find them fully as satisfactory,—rather more satisfactory in results than copper amalgam.

There is one point I did not mention. In our attempts to preserve bicuspid teeth in approximal cavities, if we have any reason to hesitate about putting in what we have spoken of as a "permanent" filling (by which term is more often meant a metal filling), I think a very good way is to use gutta-percha, perhaps half-way from the cervical wall, and then make a cement filling to the crown. That is my judgment of how to treat those cases where circumstances are not in favor of a metallic filling.

*Dr. Stevens.*—I would like to ask Dr. Bradley why his judgment leads him to do that?

*Dr. Bradley.*—My experience leads me to think that the cement is not so satisfactory at the cervical wall,—that it dissolves, or, for some reason, leaves the wall unprotected. The gutta-percha does not dissolve; it seems better adapted for the protection of the cervical wall, and yet for the crown it has not the wearing properties that the cement has, and I have had very successful results from filling those approximal cavities half-way up with gutta-percha, and then filling the crown with the best cement I can find.

William H. Potter, D.M.D.,
*Editor American Academy of Dental Science.*

